# Analyzing and validating the prognostic value and mechanism of colon cancer immune microenvironment

**DOI:** 10.1186/s12967-020-02491-w

**Published:** 2020-08-28

**Authors:** Xinyi Wang, Jinzhong Duanmu, Xiaorui Fu, Taiyuan Li, Qunguang Jiang

**Affiliations:** 1 Department of Gastrointestinal Surgery, The First Affiliated Hospital of Nanchang University, Nanchang, Jiangxi People’s Republic of China; 2 Queen Mary college, Medical Department, Nanchang University, Nanchang, Jiangxi China

**Keywords:** Colon cancer, Tumor immune microenvironment, Tumor mutational burden, Weighted correlation network analysis

## Abstract

**Background:**

Colon cancer is a disease with high malignancy and incidence in the world. Tumor immune microenvironment (TIM) and tumor mutational burden (TMB) have been proved to play crucial roles in predicting clinical outcomes and therapeutic efficacy, but the correlation between them and the underlying mechanism were not completely understood in colon cancer.

**Methods:**

In this study, we used Single-Sample Gene Set Enrichment Analysis (ssGSEA) and unsupervised consensus clustering analysis to divide patients from the TCGA cohort into three immune subgroups. Then we validated their differences in immune cell infiltration, overall survival outcomes, clinical phenotypes and expression levels of HLA and checkpoint genes by Mann–Whitney tests. We performed weighted correlation network analysis (WGCNA) to obtain immunity-related module and hub genes. Then we explored the underlying mechanism of hub genes by gene set enrichment analysis (GSEA) and gene set evaluation analysis (GSVA). Finally, we gave an overall view of gene variants and verified the correlation between TIM and TMB by comparing microsatellite instability (MSI) and gene mutations among three immune subgroups.

**Results:**

The colon cancer patients were clustered into low immunity, median immunity and high immunity groups. The median immunity group had a favorable survival probability compared with that of the low and high immunity groups. Three groups had significant differences in immune cell infiltration, tumor stage, living state and T classification. We got 8 hub genes (CCDC69, CLMP, FAM110B, FAM129A, GUCY1B3, PALLD, PLEKHO1 and STY11) and predicted that immunity may correlated with inflammatory response, KRAS signaling pathway and T cell infiltration. With higher immunity, the TMB was higher. The most frequent mutations in low and median immunity groups were APC, TP53 and KRAS, while TTN and MUC16 showed higher mutational frequency in high immunity group.

**Conclusions:**

We performed a comprehensive evaluation of the immune microenvironment landscape of colon cancer and demonstrated the positive correlation between immunity and TMB. The hub genes and frequently mutated genes were strongly related to immunity and may give suggestion for immunotherapy in the future.

## Background

Colon cancer is one of the most malignant tumors worldwide [1]. Thanks to the progression in systemically medical treatment and surgical techniques, prognosis of patients with colon cancer has dramatically improved if they are diagnosed at early stage [2]. Prognostic prediction of patients with colon cancer mainly relies on the TNM staging system, histopathological criteria, molecular markers and tumor-cell differentiation [3]. Nowadays, accumulated studies have demonstrated the role of gene mutation status, gene expression levels and signaling pathway changes in tumor progression and malignization, but it is still a challenge to find out prognostic factors which can also provide targets for therapy [4, 5]. The viewpoint that the immune system can influence the progression of cancer has been the hotspot for study over a century. Recently, numerous evidences indicate that the tumor immune microenvironment (TIM) is of great value in predicting prognosis and evaluating therapeutic efficacy factors [6]. TIM is composed of immune cells, immune-related pathways and cytokines that secreted by immune cells. In colon cancer, there has been studies showed that adaptive immune reaction is strongly correlated with survival outcomes and recurrence, and the infiltration of different kinds of immune cells might construct a favorable or unfavorable environment for tumor cells to proliferate and metastasize [7].

Since immune checkpoint-inhibiting agents, such as programmed death-1 receptor (PD1) and cytotoxic T-lymphocyte antigen 4 (CTLA-4) inhibitors, have been developed as antitumor drugs, immunotherapy has become a promising field of cancer treatment and demonstrated its impressive clinical value in patients across multiple types of solid tumors [8, 9]. Lymphocyte activation gene-3 (LAG3) is another immunotherapy target in the clinic, whose up-regulation is required to prevent the onset of autoimmunity. Sustained antigen exposure in the TIM leads to up-regulated LAG3 expression, resulting in exhaustion of immune cell proliferation and cytokine production [10]. Recently, many studies proved that the expression levels of Indoleamine 2, 3-dioxygenase 1 (IDO1) plays an important role in engender immune tolerance and pathogenic inflammatory processes, which highlights its strong association with T-cell infiltration [11]. The essence of tumor immunotherapy is to arouse and strengthen the immune system to kill tumor cells in various ways. Tumor mutational burden (TMB) was defined as the total amount of coding errors of somatic genes, base substitutions, insertions or deletions detected across per million bases [12]. If TMB is larger, the cancer cell is more mutated, and it is easier for immune cells to recognize and kill it [13]. And tumors which respond to immune checkpoint-inhibiting agents have a higher level of immune cell infiltration and exhibit a T-cell inflamed phenotype. There is a certain correlation between TIM and TMB, and exploring this correlation is of great significance for us to select immunotherapeutic drugs and explore new immunotherapeutic targets [14]. KRAS and BRAF mutational status have been considered as prognostic factors in colon cancers with MSI and may give clues for adjuvant therapy in the future [15–17]. Lin et al. have reported that activation of STAT3 plays a significant role in increasing infiltration with CD8^+^ lymphocytes and inhibiting the recruitment of T-regs that enhance colon tumor progression and immune escape [18]. There were several researches which explored the characteristics of TIM in pan-cancer and evaluate the correlation between the landscape of TIM and prognosis of patients, but they were focused on comparing different cancer types in immune cell infiltrating. As a result, further exploration of TIM in genetic level is of great significance.

In this study, by applying unsupervised consensus clustering analysis, we divided patients from the TCGA cohort into three groups (high, median and low immunity) according to ssGSEA scores. Furthermore, we validated their differences in immune cell infiltration, overall survival outcomes, clinical phenotypes and expression levels of HLA and checkpoint genes. In order to screen out essential genes for constructing colon cancer immune microenvironment, we performed WGCNA and got 8 hub genes which were in the module correlated with immune capacity. Finally, we explored the underlying mechanism of hub genes by GSEA and GSVA, and verified the correlation between TIM and TMB to give ideas for immunotherapy of patients with colon cancer.

## Methods

### Data download

The transcriptome data, somatic mutation data and clinical information of colon cancer patients were obtained from the TCGA database via the GDC data portal (https://portal.gdc.cancer.gov/repository). We downloaded RNA-seq (level 3, HTSeq-FPKM data) of 445 colon cancer patients [445primarytumortissueand41solidnormaltissue] with complete clinical information from the TCGA database. The clinical information of patients from TCGA database are summarized in Table 1. We downloaded “Masked Somatic Mutation” subtype of somatic mutation data and used the VarScan software to process it. We used a R package called “maftools” [19] to analyze and visualize the Mutation Annotation Format (MAF) of somatic variants. Human Protein Atlas (https://www.proteinatlas.org) was used to validate expression levels of hub genes by immunohistochemistry. MSI information (MSI‑H, MSI‑L or MSS) for each TCGA samples were obtained from a previous study by Liu et al.[20]. The annotations of genes were obtained from Uniprot database (https://www.uniprot.org/).

**Table 1:** Clinical characteristics of the included TCGA dataset

Characteristics		Total TCGA
		N
Age years	< 60	133
	≥ 60	312
Gender	Male	212
	Female	233
T	T1	10
	T2	76
	T3	302
	T4	56
	Unknown	1
M	M0	328
	M1	61
	Unknown	56
N	N0	264
	N1	102
	N2	79
	Unknown	0
Stage	Stage I	75
	Stage II	174
	Stage III	124
	Stage IV	61
	Unknown	11
Lymphatic invasion	No	245
	Yes	159
	Unknown	41
Venous invasion	No	292
	Yes	95
	unknown	58
Fustat	Alive	351
	Dead	94

### Implementation of Single-Sample Gene Set Enrichment Analysis ssGSEA

We obtained the marker gene sets for immune cells and immune pathways from another article [21]. We performed ssGSEA to derive the enrichment score of each immune-related term using a R package called “GSVA” [22]. The ssGSEA applies gene signatures expressed by immune cell populations and immune pathways to every cancer samples. The computational approach used in our study included immune cells types and immune pathways that are involved in innate immunity and adaptive immunity. We obtained 29 immune gene sets from several literatures, including immune cell types and functions [23], tumor-infiltrating lymphocytes (TILs) [24], proinflammatory [25], para-inflammation (PI) [26], cytokine and cytokine receptor (CCR) [27], human leukocyte antigen (HLA) [28], regulatory T (Treg) cells [29], immune checkpoint [30].

### Identification of immune subgroups by consensus clustering

To investigate the correlation between immunity and clinical phenotypes in colon cancer, we clustered colon cancer samples from TCGA into 3 different groups (high, medium and low immunity) with “Consensus Cluster Plus” (50 iterations, resample rate of 80%) based on enrichment scores of immune terms in ssGSEA. In order to validated that those 3 subgroups are different in immunity, we use a R package called “estimate” to calculate the immune score, stromal score and ESTIMATE score of every tumor sample [31]. And we compared tumor purity of samples in 3 subgroups by Mann–Whitney U test.

### Analysis of clinical information and immunotherapy-related genes

The Chi-square test was performed to analyze the correlation between immunity and clinical phenotypes, including gender, age, venous invasion, lymphatic invasion, stage, TNM classification and survival state. We classified the total TCGA cohort into subgroups based on clinical phenotypes: gender (male/female), age (> 60/ ≤ 60), venous invasion (yes/no), lymphatic invasion (yes/no), stage (stage1 + stage2/stage3 + stage4), T(T1 + T2/T3 + T4), N(N0/N1 + N2), M(M0/M1). And we analyzed the difference in overall survival rate between 3 immune subgroups in clinical subgroups by a R package called “survival”. The expression level of human leukocyte antigens (HLA) and checkpoint-related genes in 3 immune subgroups were compared by Mann–Whitney test. The proportions of the 22 tumor infiltrating immune cells in 3 immune subgroups were determined by Kruskal–Wallis tests using a R package called “CIBERSORT” [32].

### Construction of co-expression module networks

The Weighted correlation network analysis (WGCNA) was used to construct the gene co-expression network to find clinical-phenotype-related modules and hub genes by the R package “WGCNA”[33]. All genes and samples were filtered by good genes or good samples test. Filtered genes were used to construct a scale-free network by calculating the connection strength between genes. Scale-free R^2^ ranging from 0 to 1 was used to determine a scale-free topology model. To minimize effects of noise and spurious associations, the adjacency matrix was transformed into Topological Overlap Matrix (TOM). And TOM-based dissimilarity was used to form modules by dynamic tree cut. Here, we set minimal module size as 30 and cut height as 0.25. We evaluated the correlation among module eigengenes (MEs), clinical traits and modules which are related to the traits. For each module, gene significance (GS) and module membership (MM) were calculated and used for hub gene selection. Moreover, Kyoto Encyclopedia of Genes and Gene Ontology (KEGG) pathway enrichment analyses and Gene Ontology (GO) analysis were performed for genes in the modules using the KOBAS database. The cutoff criteria set as p value < 0.05.

### Predicting underlying mechanism of immunity-related modules and hub genes

Wilcox test was performed to compare the expression level of hub genes in normal samples colon cancer samples with different clinical phenotypes. We performed GSEA [22] and GSVA to explore correlated pathways of our immune-related risk signature. Gene ontology gene sets “h.all.v7.0.symbols.gmt” were downloaded from Molecular Signatures Database (MSigDB, https://software.broadinstitute.org/gsea/downloads.jsp) and were used for the enrichment analysis. When the false discovery rate (FDR) was less than 0.25, the enriched gene set was considered to be statistically significant. We demonstrated the correlation between hub genes expression levels and immune cells infiltration by calculating the Person correlation coefficients, which was performed by using TIMER database (https://cistrome.shinyapps.io/timer). The function of hub-genes was analyzed by Metascape database (http://metascape.org/) [34].

### Calculation of TMB scores and prognostic analysis

In our study, we calculated the mutation frequency with number of variants/the length of exons for each sample via Perl scripts based on the JAVA8 platform. We classify the colon cancer samples into low-TMB and high-TMB groups according the median data. Mann–Whitney test was conducted to compare the TMB difference among 3 immune subgroups. The survival curves for the prognostic analysis were generated via the Kaplan–Meier method and log-rank tests were utilized to identify significance of differences.

## Results

### Immune microenvironment landscape of colon cancer

**Fig. 1:**
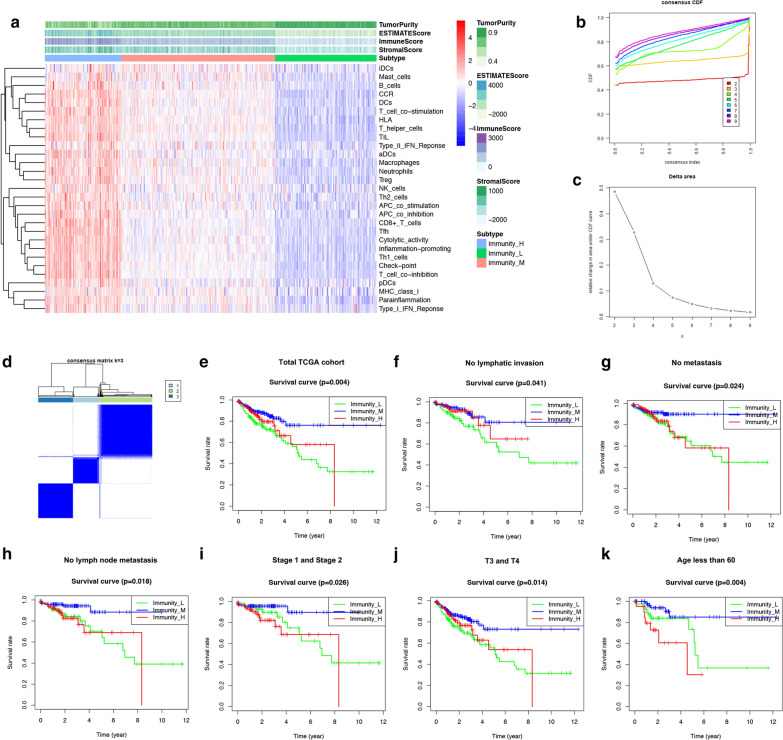
Identification and validation of colon cancer immunity-related subgroups. **a** In ssGSEA, 29 immune-related gene sets are enriched with colon cancer. These gene sets are composed of immune cells and immune processes. The tumor purity, ESTIMATE score, immune score and stromal score are also shown in this heatmap. **b** Consensus clustering cumulative distribution function (CDF) for k = 2 to 9. **c** Relative change in area under CDF curve for k = 2 to 9. **d** Heatmap of sample clustering at consensus k = 3. **e**–**k** Survival analysis of the total TCGA cohort, samples without lymphatic invasion. samples without metastasis, samples without lymph node metastasis, samples which are stage1 or 2, samples which are T3 or T4 and samples with age less than 60

The immunity of tumor samples was assessed by applying the ssGSEA approach to the transcriptomes of TCGA colon cancer samples. 29 immune-related pathways and infiltrating immune cells were incorporated to estimate the immune capacity of colon cancer tissues (Fig. 1a). The total TCGA cohort were clustered into 3 subgroups (low immunity: 136 samples, median immunity: 206 samples, high immunity: 103 samples) by applying unsupervised consensus clustering analysis (Fig. 1b–d). To validate the immunity of 3 immune subgroups, we also showed the ESTIMATE score, immune score and stromal score in the heatmap. The association of immunity and colon cancer patients’ prognosis was indicated by comparing survival rates of 3 immune subgroups in different clinical subgroups (Fig. 1e–k, Additional file 1: Figure S1). The result showed that survival rates of 3 immune groups have statistical difference in the total TCGA cohort (P = 0.004), age < 60 (P = 0.019), no lymphatic invasion (P = 0.041), M0 (P = 0.024), N0 (P = 0.018), stage1 + stage2 (P = 0.026) and T3 + T4 (P = 0.014). In all of these clinical subgroups, patients with median immunity have the best prognosis while patients with lowest immunity have the worst prognosis. The Chi-square test (Additional file 2: Figure S2) showed that immunity classification was correlated with stage (P < 0.001), metastasis (P < 0.001), N classification (P < 0.01) and survival state (P < 0.05). This demonstrated that immunity could have strong correlation with clinical phenotypes and also serve as a prognostic factor in colon cancer.

### immune subgroups are different in immune cell infiltration and expression of immunotherapy-related genes

**Fig. 2:**
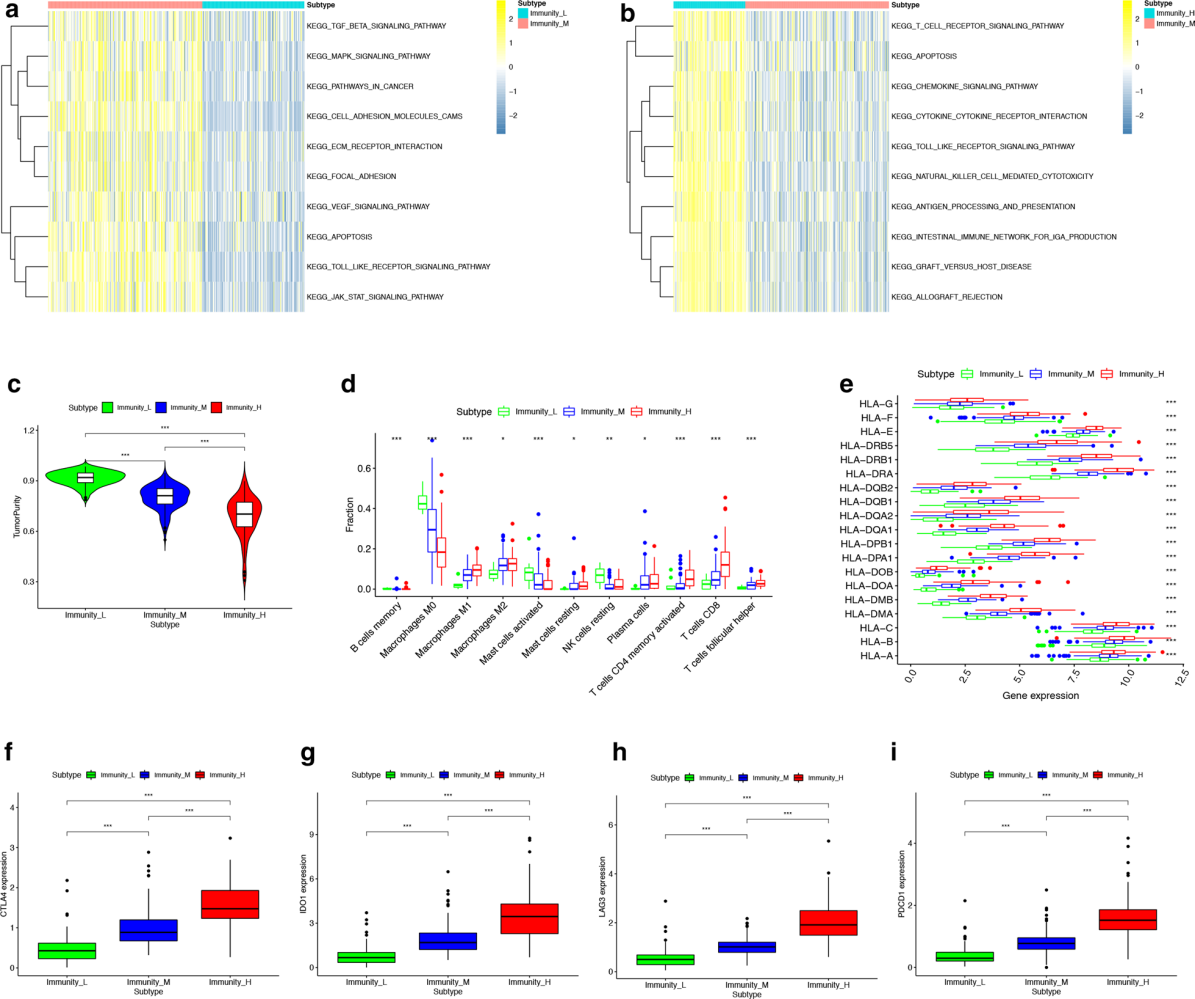
GSVA and analysis of immune cell infiltration, HAL genes and checkpoint genes expression in 3 immune subgroups. **a, b** The heatmap was used to visualize these biological processes, and yellow represented activated pathways and blue represented inhibited pathways. The colon cancer cohorts were used as sample annotations. A: Immunity-L vs Immunity-M, B: Immunity-M vs Immunity-H. **c** The tumor purity of samples from 3 immune subgroups (*P < 0.05, **P < 0.01, ***P < 0.001). **d** The fractions of 11 types of infiltrating immune cells in samples from 3 immune subgroups. **e** The RNA expression levels of HLA genes in samples from 3 immune subgroups. **f, i** The RNA expression levels of checkpoint-related genes (CTLA4, IDO1, LAG3 and PDCD1) in samples from 3 immune subgroups

To explore the biological behaviors among these immune subtypes, we performed GSVA enrichment analysis. As shown in Fig. 2a, b, Immunity-L was related to immune suppression biological process. Immunity-M was enriched in stromal and carcinogenic activation pathways such as TGF beta signaling pathway, apoptosis, VEGF and MAPK signaling pathways. Immunity-H was associated with immune activation including the activation of chemokine signaling pathway, cytokine-cytokine receptor interaction, T cell receptor signaling pathway and Natural killer cell mediated cytotoxicity. These 3 immune subgroups were also significantly different in tumor purity: the high immunity group has the lowest tumor purity and the low immunity group has the highest tumor purity (Fig. 2c). The fraction of 20 types of infiltrating immune cells were compare among 3 immune subgroups (Fig. 2d). The result showed that 11 types of immune cells, including B cell, macrophages M1, macrophages M2, macrophages M3, resting mast cell, activated mast cell, NK cell, plasma cell, CD4 T cell, CD8 T cell and T cell follicular helper, had significantly different infiltrating levels in different immune subgroup. The expression level of 19 HLA genes were all significantly different among 3 immune subgroups (Fig. 2e). With higher immunity, the expression level of HLA genes was higher. We chose 4 immune checkpoint genes which are regarded as targets in immunotherapy, including CTLA4, IDO1, LAG3 and PDCD1(PD-1). And we found that the expression level of all these genes are highest in high immunity group and lowest in low immunity group (Fig. 2f–i). Median immunity group has medium expression level of those 4 genes. As a result, patients in high immunity group may be more sensitive to immune checkpoint-inhibiting agents, such as PD1 inhibitors and CTLA-4 inhibitors. The different landscape of immune cell infiltrating could also give ideas for immunotherapy, as high immunity group has higher level of T cells (CD4 T cell, CD8 T cell and T cell follicular helper) infiltration while low immunity group has higher level of macrophages M0, mast cell and NK cell infiltration.

### Detection of immunity-related module and 8 hub genes by WGANA

**Fig. 3:**
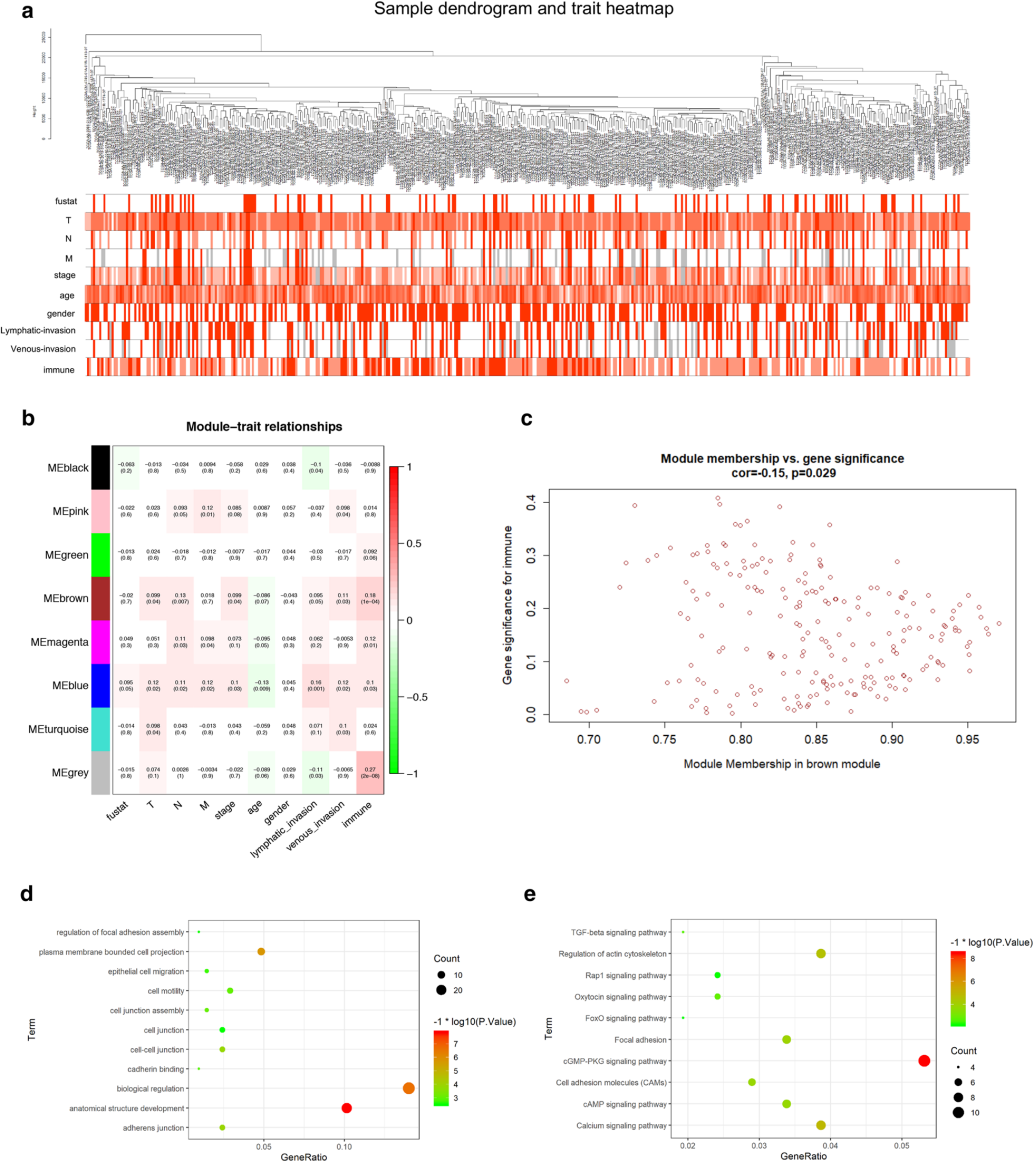
Detection and validation of immunity-related module by WGCNA. **a** The cluster was based on the transcriptome data from TCGA. The color intensity represents the clinical phenotypes (fustat, TNM classification, stage, age, gender, lymphatic invasion, venous invasion and immunity). **b** Heat‐map of the correlation between gene modules and the clinical phenotypes of colon cancer. The brown module was the most correlated module with immunity. **c** The correlation analysis between membership (MM) in brown module and gene significance (GS) for immunity. **d, e** Bubble chart of GO and KEGG results of brown module

In WGCNA analysis, we identified 8 co-expression modules and analyzed their association with 10 clinical phenotypes, including fustat, TNM classification, stage, age, gender, lymphatic invasion, venous invasion and immunity (high, median and low) (Fig. 3a, b, Additional file 3: Figure S3). Except the grey module which contained non-clustering genes, the brown module was the most correlated module of immunity (r = 0.18, P = 1e-04, Fig. 3c). There were 212 genes in the brown module (Additional file 4: Table S1). The brown module was also correlated with T (r = 0.099, P = 0.04), N (r = 0.13, P = 0.007), stage (r = 0.099, P = 0.04) and venous invasion (r = 0.11, P = 0.03). In the module-trait analysis, 8 genes with GS value > 0.3 and MM value > 0.8 were defined as hub genes: CCDC69, CLMP, FAM110B, FAM129A, GUCY1B3, PALLD, PLEKHO1 and STY11. The GS values and MM values of 8 hub genes were shown in Additional file 5: Table S2. These hub genes were selected for further analysis. We validated the correlation between the relative infiltrating level of immune cells and the expression level of hub genes by the TIMER database (Additional file 6: Figure S4). The result demonstrated that the expression of 8 hub genes have negative correlation with tumor purity and their expression level were positively correlated with the infiltration of CD4 T cells, macrophages, neutrophils and dendritic cells. To investigate the underlying mechanism of the immunity-related module, we performed GO and KEGG analysis (Fig. 3d, e). In GO analysis, GO terms such as biological regulation, anatomical structure development and plasma membrane bounded cell projection were enriched in the brown module. In KEGG pathway analysis, cGMP-PKG, calcium and cAMP signaling pathways are also enriched with the brown module.

### Prognosis value and underlying mechanism of hub genes

**Fig. 4:**
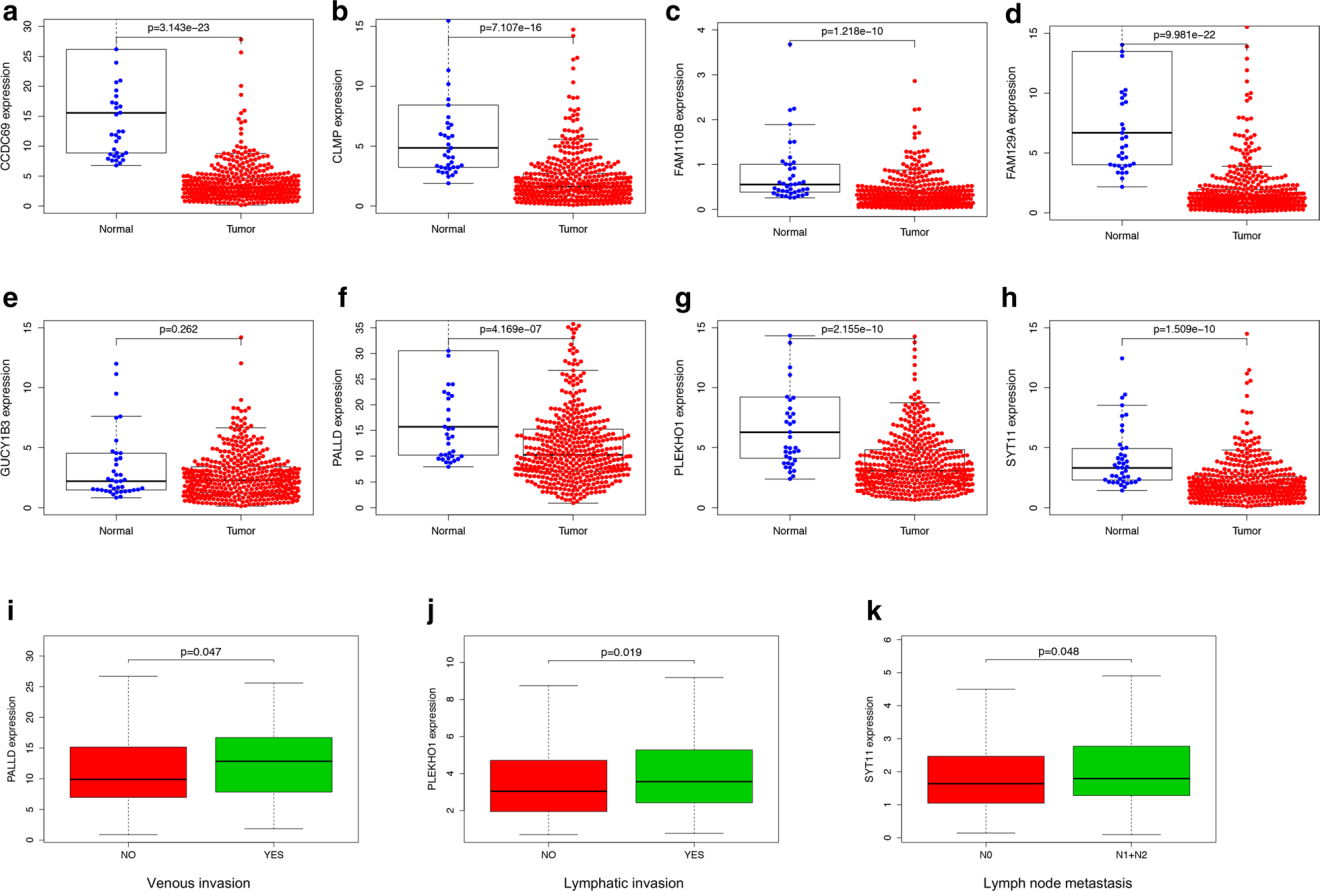
Mann–Whitney test of 8 hub genes expression in different types of samples. **a**–**h** Expression level of 8 hub genes in normal tissue and tumor tissue. **i**, **j** The expression levels of hub genes are different in clinical subgroups. PALLD was correlated with venous invasion (P = 0.047), PLEKHO1 was correlated with lymphatic invasion (P = 0.019) and SYT11 was correlated with lymph node metastasis (P = 0.048)

**Fig. 5:**
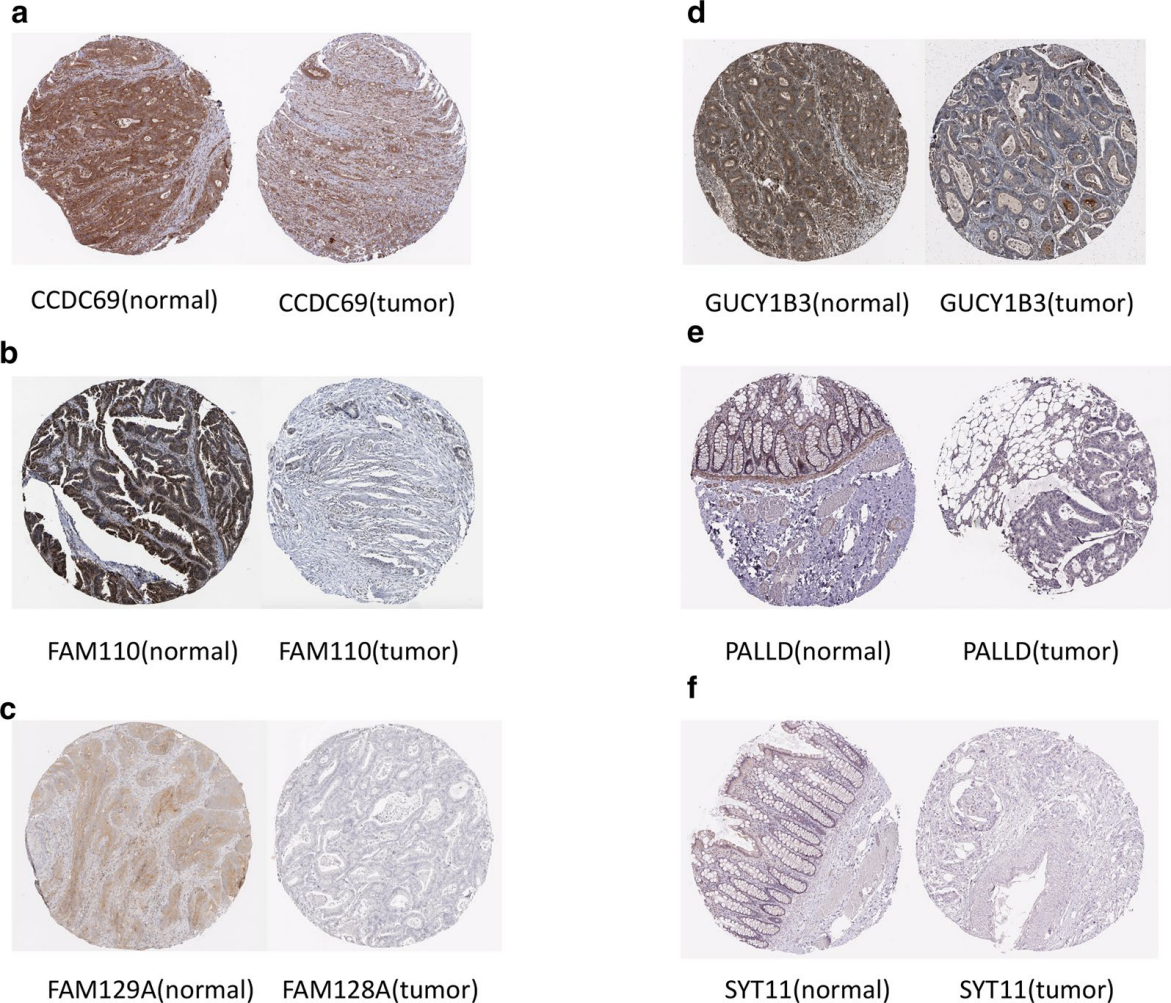
The expressional differences of hub gene levels between colon cancer tissues and the para-cancer normal solid tissues in the Human Protein Atlas database

**Fig. 6:**
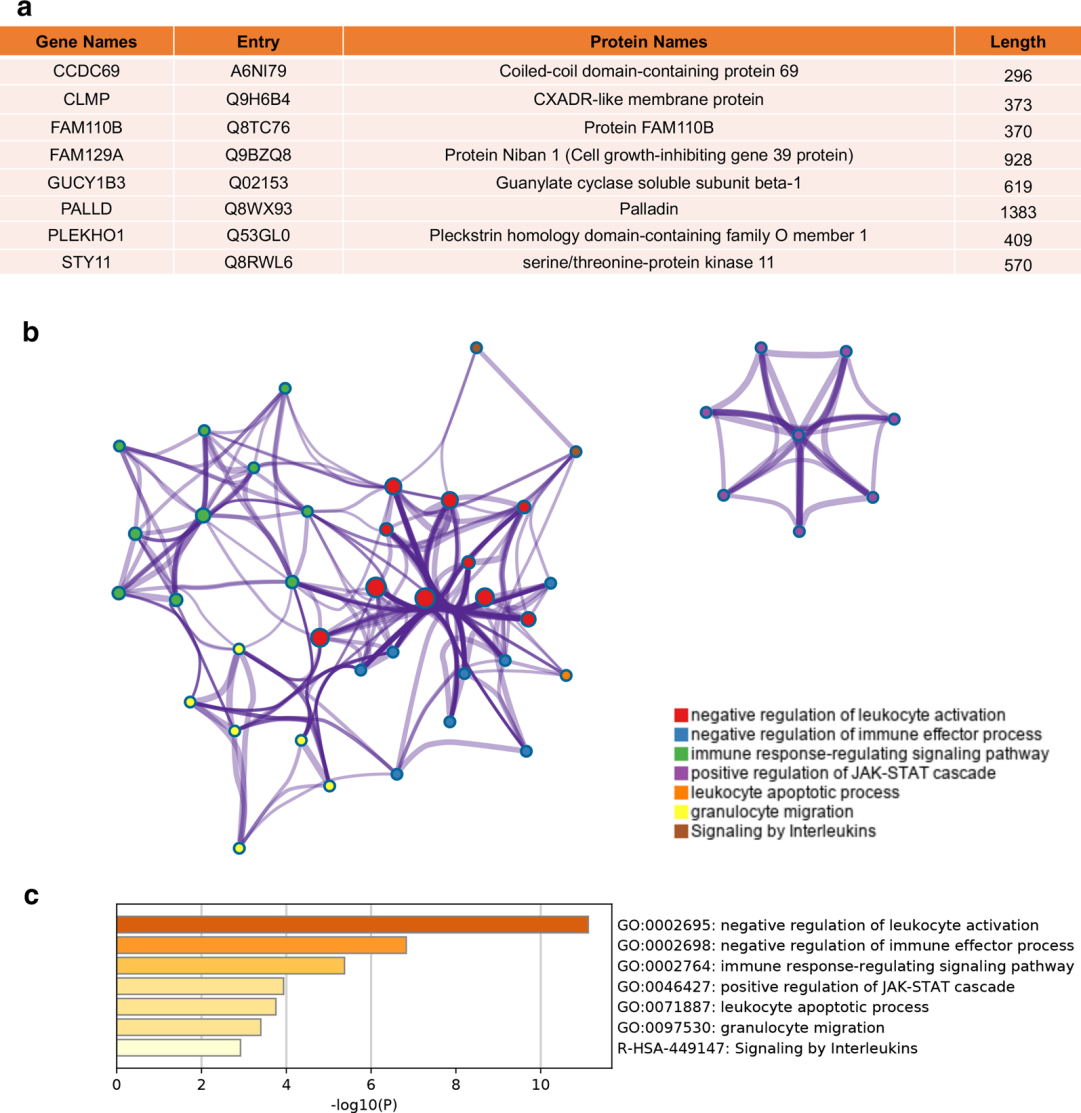
Functional analysis of hub genes. **a** The annotation of hub genes using Uniport database. **b** Protein–Protein interaction network of genes which were directly related to hub genes. A sport represented a gene and the color of spots represented which pathway this gene was involved in. **C** The enrichment statistical significance of GO-terms and KEGG pathways

Using transcriptome data from TCGA, we noticed that 7 of 8 hub genes are differentially expressed in colon cancer tissue and normal solid tissue (Fig. 4a–h). And all of them have lower expression level in cancer tissues. We validated the protein expression of these hub genes based on IHC samples provided by the Human protein Atlas database. Compared to normal tissues, 6 of 8 hub genes were over-expressed in tumor tissues (Fig. 5a–f). Clinical information analysis indicated that PALLD was correlated with venous invasion (P = 0.047, Fig. 4i), PLEKHO1 was correlated with lymphatic invasion (P = 0.019, Fig. 4j) and SYT11 was correlated with lymph node metastasis (P = 0.048, Fig. 4k). We annotated 8 hub-genes in Fig. 6a and used Metascape database to explore the function of these hub-genes (Fig. 6b, c). These genes were related to negative regulation of leukocyte activation and immune effector process, positive regulation of JAK-STAT signaling pathway, leukocyte apoptosis and granulocyte migration. Then we performed GSEA to explore the underlying mechanism of hub genes by assessing the enrichment of cancer hallmark gene sets (Additional file 7: Figure S5). The high-expression of most hub genes were enriched with epithelial mesenchymal transition, IL2-STAT signaling, IL6-JAK-STAT3 signaling, inflammatory response and KRAS signaling. Interestingly, these hallmarks are recognized to be related with immune reaction, progression of cancer and immunotherapy in some extent. In addition, the down-expression of these genes were enriched with MYC target, which was also an important oncogene target in cancer development.

### TMB landscape in colon cancer and its correlation with immunity

**Fig. 7:**
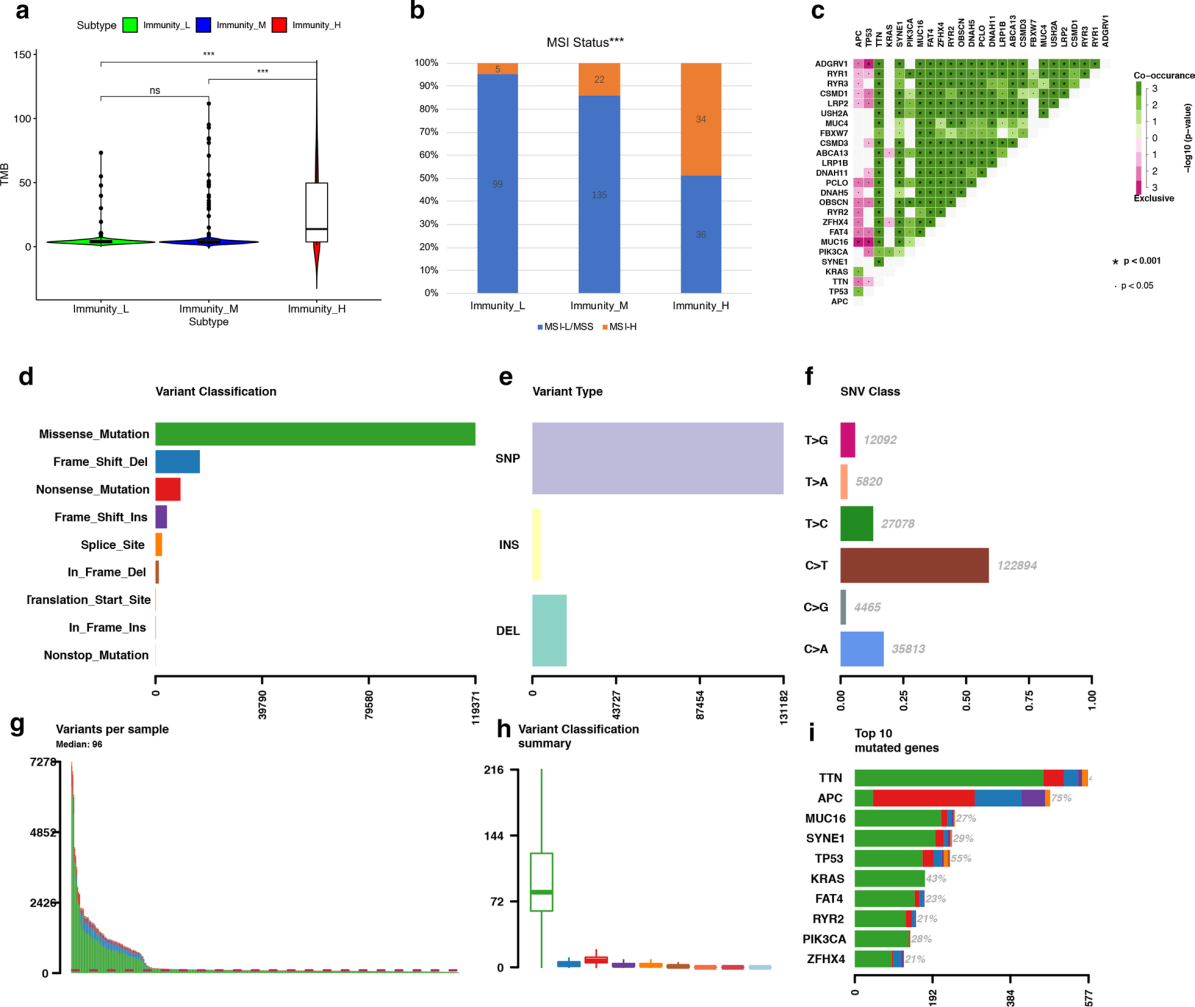
The landscape of frequently mutated genes in colon cancer. **a** The TMB of samples from 3 immune subgroups (*P < 0.05, **P < 0.01, ***P < 0.001). **b** The Chi-square test of MSI status in 3 immune subgroups. **c** The coincident and exclusive associations across mutated genes. **d** Classification and frequency of mutation types. **e** Frequency of variant types. **f** Frequency of SNV classes. **g, h** tumor mutation burden in specific samples; **i** the top 10 mutated genes in colon cancer

**Fig. 8:**
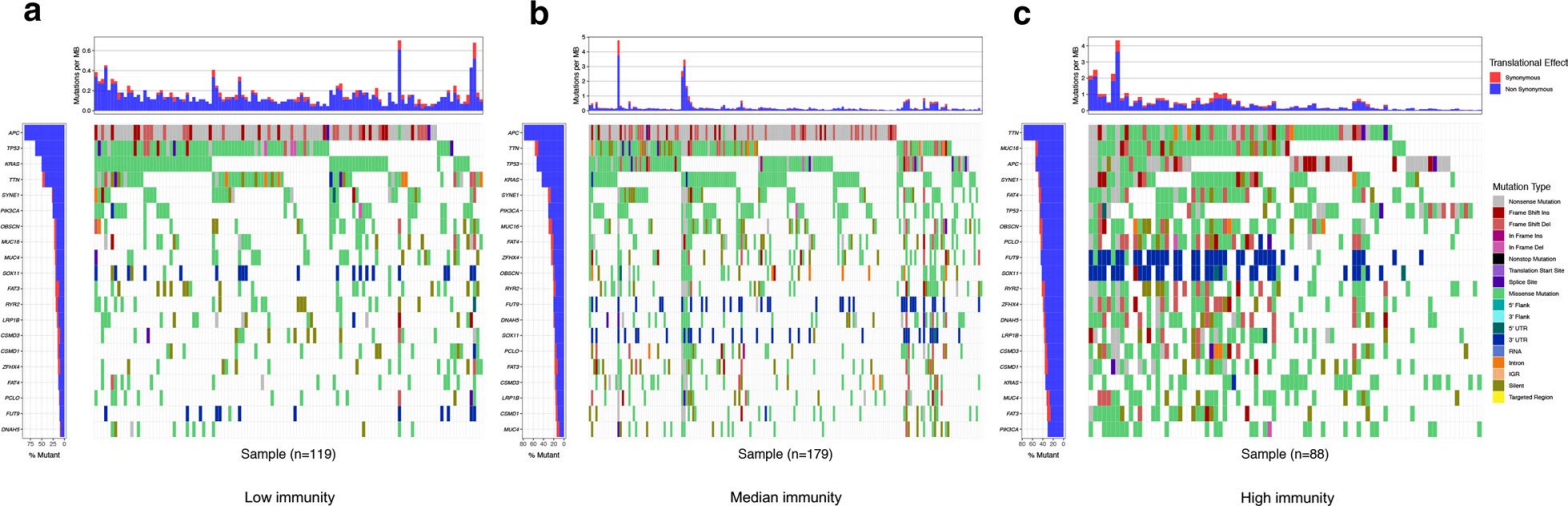
Frequently mutated genes in 3 immune subgroups. **a**–**c** Waterfall plots display the frequently mutated genes in 3 immune subgroups of colon cancer. The left panel shows the genes ordered by their mutation frequencies. The right panel presents different mutation types

The TMB of samples from 3 immune subgroups were compared by Mann–Whitney test, indicating that tumors with higher immunity have higher TMB (Fig. 7a). The MSI status (proportion of MSI-H and MSI-L/MSS) were compared among 3 immune subgroups by Chi-square test (Fig. 7b). The exclusive and coincident associations across mutated genes were shown in Fig. 7c. These mutations were further classified into different categories: missense mutation, delectation, nonsense mutation, splice site, insertion, translation start site and nonstop mutation (Fig. 7d). For variant types, single nucleotide polymorphism (SNP) had a higher frequency than insertion or deletion (Fig. 7e), and C > T was the most common single nucleotide variants (SNV) (Fig. 7f). Besides, we counted the number of altered bases in each sample and showed mutation types in box plot (Fig. 7g–h). Finally, we exhibited the top 10 mutated genes in colon cancer with ranked percentages, including TTN (47%), APC (75%), MUC16 (27%), SYNE1 (29%), TP53 (55%), KRAS (43%), FAT4 (23%), RYR2 (21%), PIK3CA (28%) and ZFHX4 (21%) (Fig. 7i). Muation information of each sample in 3 immune subgroups (low immunity: n = 119, median immunity: n = 179, high immunity: n = 88) was exhibited in waterfall plot (Fig. 8a–c). We founded that the proportion of samples with specific mutated genes was different among 3 immune subtypes, which may provide suggestion for clinical application of immunotherapy.

## Discussion

For colon cancer, patients with same clinical phenotypes can have different prognosis. As the relationship between chronic inflammation and colon cancer had been well demonstrated, more and more people began to study the role of immunity in cancer progression and considered it as one possible prognostic factor. In this study, we depicted the immune landscape of colon cancer using transcriptome and clinical information downloaded from the TCGA database. The colon cancer samples were clustered into three clusters (low immunity, median immunity and high immunity). The patients in the median-immunity cluster had the best prognosis compared with patients in the low-immunity and high-immunity clusters. Patrick Danaher et al. established a tumor inflammation signature based on 30 types of cancers and found that high immunity was related to better prognosis in skin cutaneous melanoma and sarcoma, while low immunity was related to better prognosis in pancreatic adenocarcinoma and lower grade glioma. These findings were different from ours, which demonstrated heterogeneity in immune landscape among different cancers. Generally, cytotoxic T cell (CD8 + T cell) immune response is considered to have anti-tumor effects by IFN-γ, TNF-α and IL17. As a result, increased T cell infiltration in tumor tissue may lead to an anti-tumor effect in the high-immunity group. But in our study, the patients in the median immunity cluster had the best prognosis compared with patients in the low immunity and high immunity clusters. Robert D. et al. concluded that the immunity mainly plays three roles in anti-tumor effects: prevents the establishment of inflammatory, protects the host from viral infection, kills tumor cells in specific tissue. However, cytokines such as IL-12 and interferon-γ (IFN-γ) can contribute to the construction of immunoediting and immune escape [35]. Convincingly, immunity can also provide the selective pressure that accelerates the proliferation of tumor cells which have gained immune-evasive mutations [36]. Our TMB analysis showed that the high immunity cluster had the highest mutational burden which also provide evidence for the establishment of immune-evasive mutations and further immune escape. So, it is arbitrary to conclude that patient with higher immunity can have better prognosis. This finding could explain that patients who keep an equilibrium between immune elimination and immunoediting may have better prognosis.

By using WGCNA, we obtained 8 hub genes which occupied important positions in the immune mechanism of colon cancer. These hub genes had lower transcriptional expression levels in tumor tissue than normal tissue. In addition, by analyzing transcriptome data from TCGA, the RNA translational levels of seven hub genes have statistical difference in tumor and normal tissues. FAM110B has been proved to have an essential role in multiple cancer hallmarks and progression of many types of cancer such as prostate cancer [37]. FAM129 can affect invasion and proliferation by regulating autophagy, unfolded protein response and FAK signaling pathway [38]. The differential expression of GUCY1B3 has also been detected in breast cancer and ovarian cancer and though to inhibit tumor angiogenesis [39]. Ma et al. reported that long noncoding RNA DUXAP8 can promote tumor progression by silencing PLEKHO1, revealing the anti-tumor effect of PLEKHO1 expression [40]. In GO and KEGG analysis, we noticed that immunity-related module had strong correlation with anatomical structure development and plasma membrane bounded cell projection. CLMP has been known as a new component of epithelial tight junctions, which support the function of hub genes in tumor cell metastasis [41]. And the result from Metascape database also proved that 8 hub-genes can have negative regulation on immune system. In further study, we need to perform deeper exploration in mechanisms and biofunctions of these hub genes, as their relationships with colon cancer have been seldomly reported.

The result of GSEA and TMB analysis indicated that high immunity was correlated with KRAS signaling pathway and high frequency of KRAS gene mutation. The expression levels of HLA genes and checkpoint genes (PD1/PDL1, CTLA4, IDO1 and LAG3) are higher in high immunity subgroup than median and low immunity subgroups, which convinced that our classification strategy were capable to provide support for immunotherapy. It has been reported that KRAS mutations could predict the resistance to epidermal growth factor receptor (EGFR) inhibitors such as cetuximab [42]. The prognostic value of KRAS mutations may be influenced by many factors, including primary tumor site, tumor stage, and adjuvant treatment received [21, 43]. Besides, stage 2 colon cancer patients with KRAS mutation were also reported to have increased risk of recurrence which was not affected by adjuvant chemotherapy. In the TMB analysis, classical tumor-related genes APC, TTN and TP53 also showed high mutational frequency among 3 immune subgroups. It is convinced that either polyposis or nonpolyposis syndromes can contribute to the genetic vulnerability to colon cancer, which is associated with mutation or loss of APC gene and several DNA mismatch repair genes [44, 45]. Xingyu Cheng et al. suggested that TTN and TP53 double mutation may participate in tumorigenesis by regulating downstream pathways with the participation of other co-expressed genes on the signaling network [46]]. In the future, it is of great significance to apply highly mutated genes and their correlated signaling pathways to searching for new targets for immunotherapy.

## Conclusion

In this study, we divided patients from the TCGA cohort into three immune subgroups (high, median and low immunity) by applying unsupervised consensus clustering analysis. Three groups were different in survival outcome, stage, metastasis, lymph node metastasis, immune cell infiltration and expression levels of HLA and checkpoint genes. Then we performed WGCNA and got 8 hub genes (CCDC69, CLMP, FAM110B, FAM129A, GUCY1B3, PALLD, PLEKHO1 and STY11), which were in the module correlated with immune capacity. In functional analysis, we found that immunity was related to signaling pathways, such as inflammatory response and KRAS signaling pathway. Finally, we indicated that immunity was positively correlated with TMB and the mutational frequency of genes were significantly different among 3 immune subgroups.

## Supplementary information

**Additional file 1: Figure S1.** Survival analysis of colon cancer samples in different clinical subgroups. **(A-H) **Comparation of overall survival rate of 3 immune subgroups in different clinical subgroups (venous invasion, female, male, lymphatic invasion, M1, N1+N2, stage3+stage4, T1+T2). In all of these subgroups, there were no statistical differences among 3 immune subgroups in survival rate.

**Additional file 2: Figure S2.** Correlation between immunity and clinical phenotypes. The Chi-square test was performed to analyze the correlation between immunity (low, median and high) and clinical phenotypes (fustat, TNM classification, stage, age, gender, lymphatic invasion, venous invasion and immunity). We found that immunity was correlated with fustat, stage, M and N (* P<0.05, ** P<0.01, *** P<0.001).

**Additional file 3: Figure S3.** WGCNA analysis of colon cancer based on TCGA transcriptome data. **(A) **Hierarchical cluster analysis was performed to detect co-expression modules with corresponding colors. **(B-C)** Soft-thresholding power analysis was used to obtain the scale-free fit index of network topology.

**Additional file 4: Table S1** The list of genes in the brown module.

**Additional file 5: Table S2** The GS and MM values of hub genes.

**Additional file 6: Figure S4** Validating the correlation between hub genes and immune cell infiltration. **(A-H)** We use the TIMER database to validate the correlation between the expression level of hub genes and the infiltration level of B cells, CD8+ cells, CD4+ cells, macrophages, neutrophils and dendritic cells in colon cancer tissues. The coefficient values and P values were calculated by Spearman coefficient.

**Additional file 7: Figure S5** GSEA of hub genes. **(A-H) **The high-expression of most hub genes were enriched with epithelial mesenchymal transition, IL2-STAT signaling, IL6-JAK-STAT3 signaling, inflammatory response and KRAS signaling. The down-expression of these genes were enriched with MYC target V1 and V2.

## Data Availability

All analyzed data are accessible online, and the results of this article are included within the article as well as in additional files.

## Abbreviations

TIM: Tumor immune microenvironment
TMB: Tumor mutational burden
ssGSEA: Single-Sample Gene Set Enrichment Analysis
WGCNA: Weighted correlation network analysis
TIM: Tumor immune microenvironment
PD1: Programmed death-1 receptor
CTLA-4: Cytotoxic T-lymphocyte antigen 4
LAG3: Lymphocyte activation gene-3
IDO1: Indoleamine 2, 3-dioxygenase 1
MAF: Mutation Annotation Format
HLA: Human leukocyte antigens
TOM: Topological Overlap Matrix
MEs: Module eigengenes
GS: Gene significance
KEGG: Kyoto Encyclopedia of Genes and Gene Ontology
GO: Gene Ontology
GSEA: Gene set enrichment analysis
MSigDB: Molecular Signatures Database
FDR: False discovery rate
SNP: Single nucleotide polymorphism
SNV: Single nucleotide variants
IFN-γ: Interferon-γ
