# Supplementary figures and images for: Analyzing and validating the prognostic value and mechanism of colon cancer immune microenvironment
Source: J Transl Med. 2020 Aug 28;18:324. doi: 10.1186/s12967-020-02491-w (PMC7456375; doi:10.1186/s12967-020-02491-w)

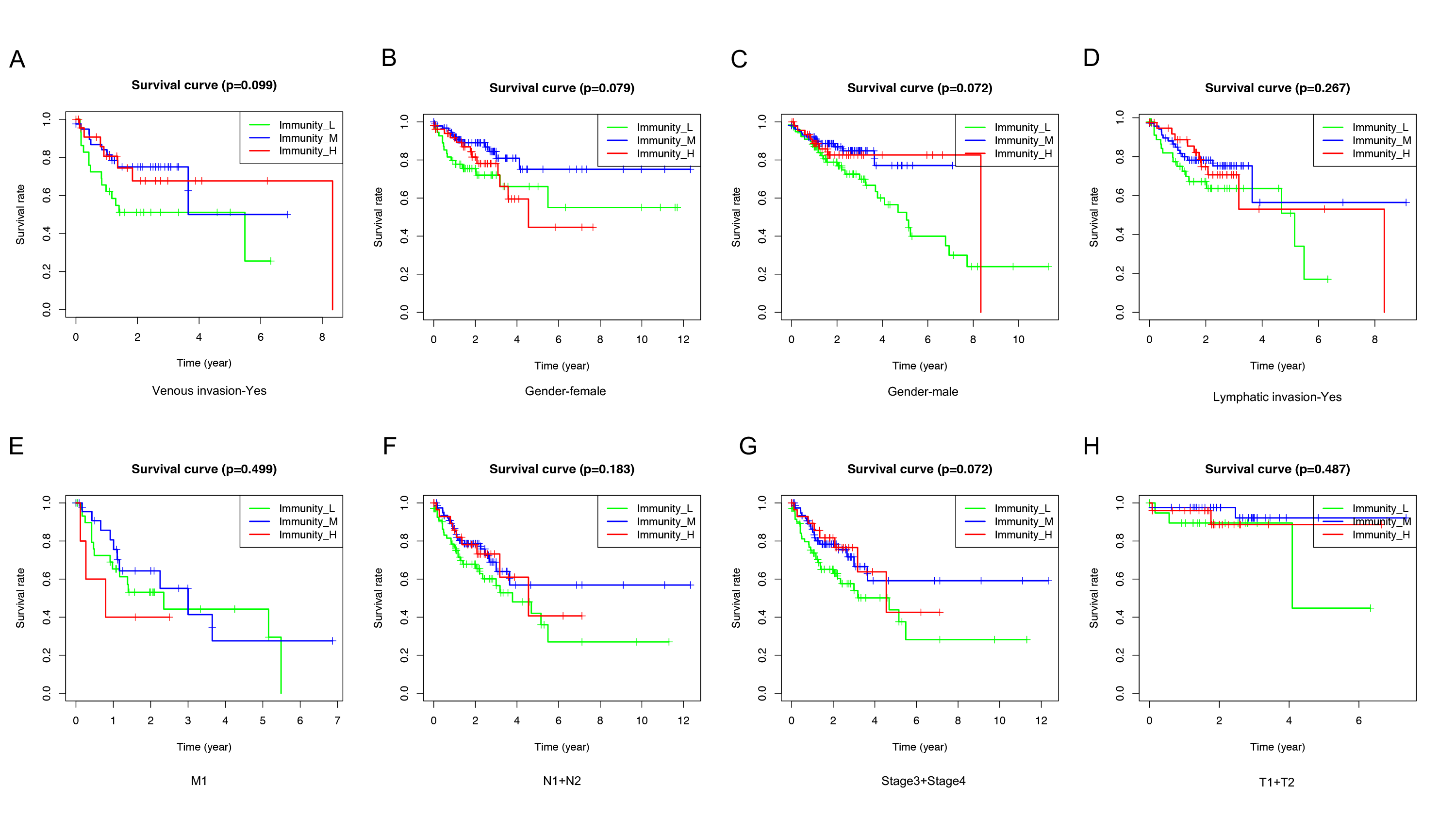

Supplement: Supplementary file 1 — Additional file 1: Figure S1. Survival analysis of colon cancer samples in different clinical subgroups. (A-H) Comparation of overall survival rate of 3 immune subgroups in different clinical subgroups (venous invasion, female, male, lymphatic invasion, M1, N1+N2, stage3+stage4, T1+T2). In all of these subgroups, there were no statistical differences among 3 immune subgroups in survival rate. [file 12967_2020_2491_MOESM1_ESM.tif]

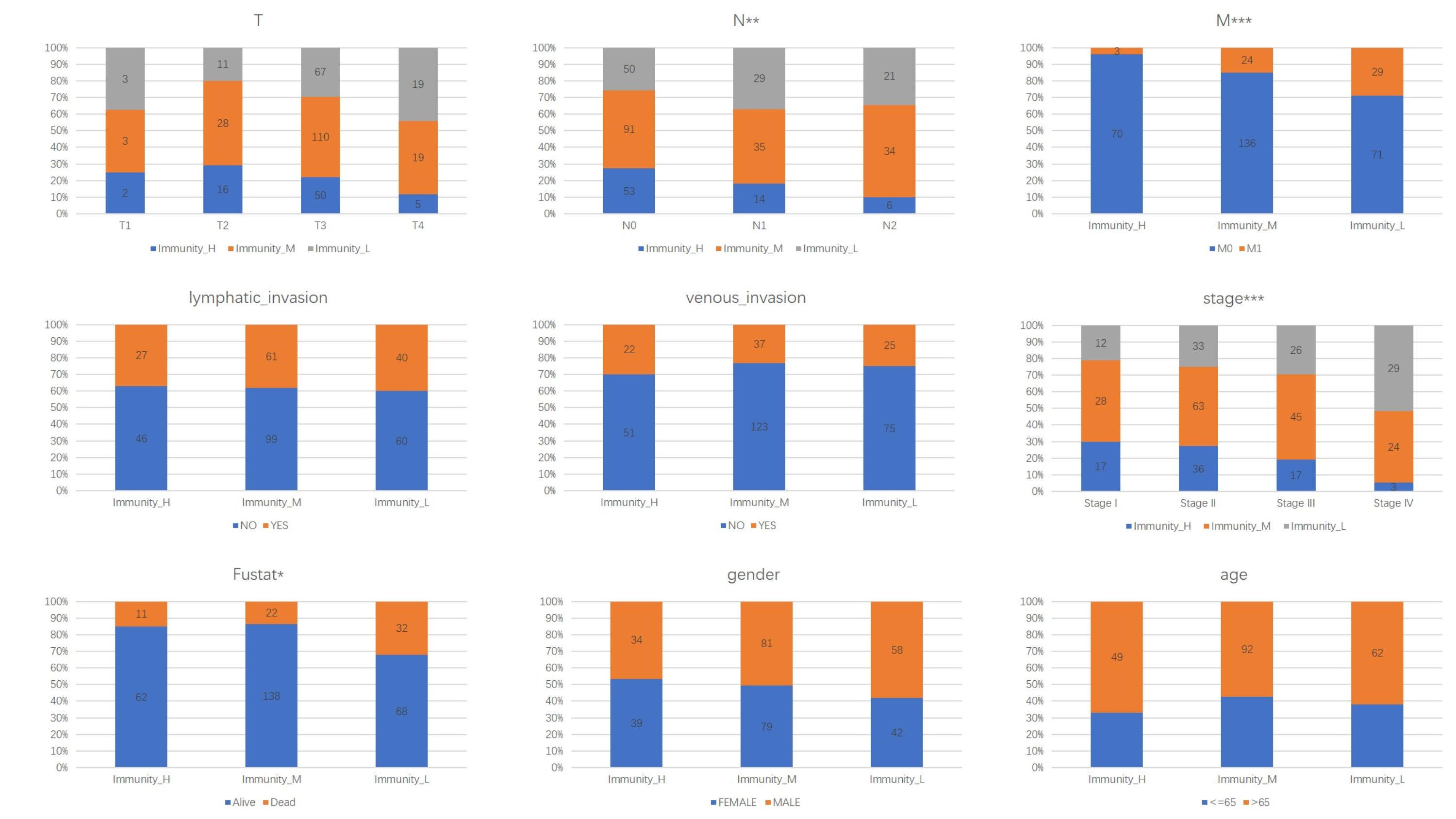

Supplement: Supplementary file 2 — Additional file 2: Figure S2. Correlation between immunity and clinical phenotypes. The Chi-square test was performed to analyze the correlation between immunity (low, median and high) and clinical phenotypes (fustat, TNM classification, stage, age, gender, lymphatic invasion, venous invasion and immunity). We found that immunity was correlated with fustat, stage, M and N (* P<0.05, ** P<0.01, *** P<0.001). [file 12967_2020_2491_MOESM2_ESM.tif]

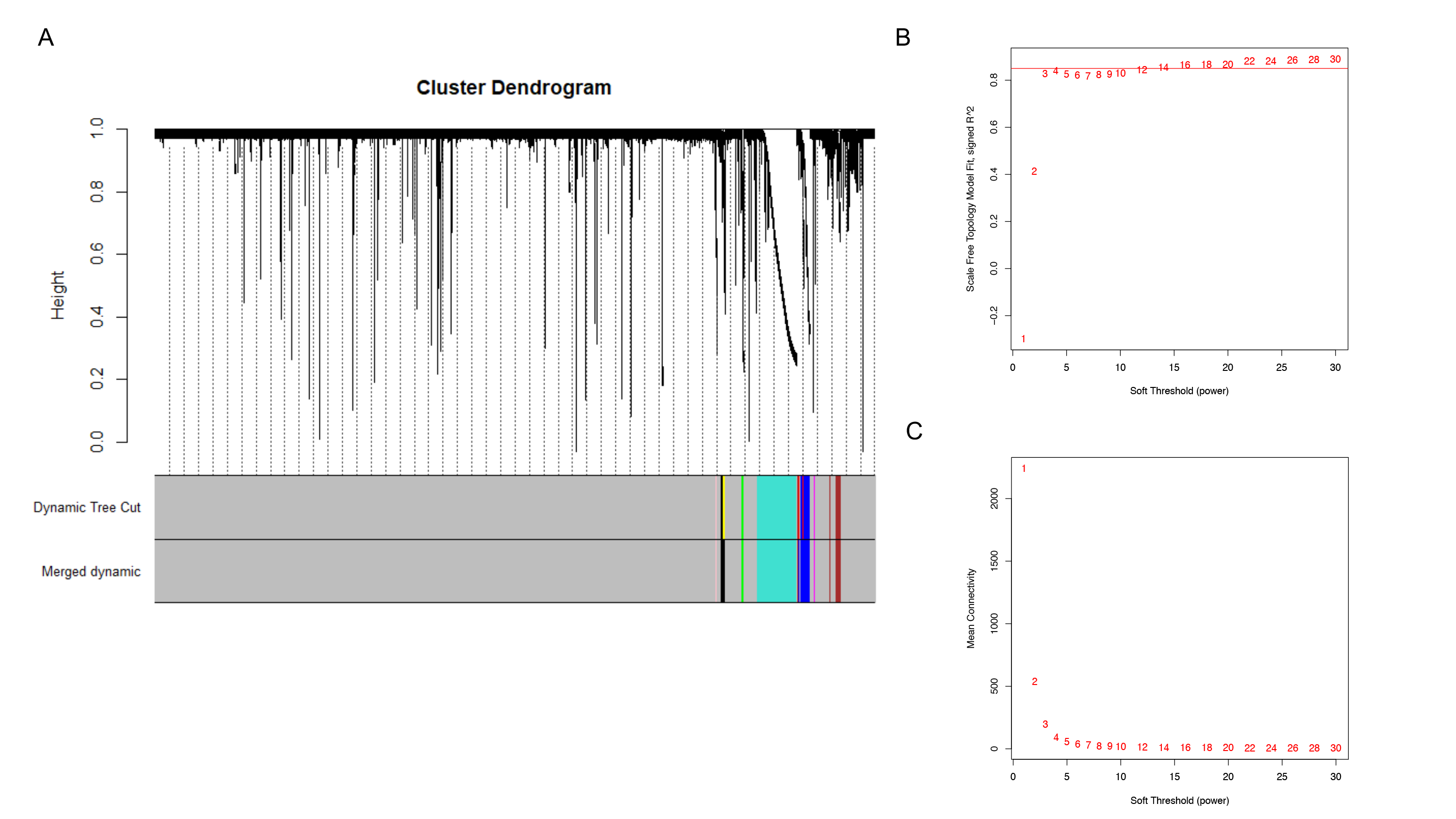

Supplement: Supplementary file 3 — Additional file 3: Figure S3. WGCNA analysis of colon cancer based on TCGA transcriptome data. (A) Hierarchical cluster analysis was performed to detect co-expression modules with corresponding colors. (B-C) Soft-thresholding power analysis was used to obtain the scale-free fit index of network topology. [file 12967_2020_2491_MOESM3_ESM.tif]

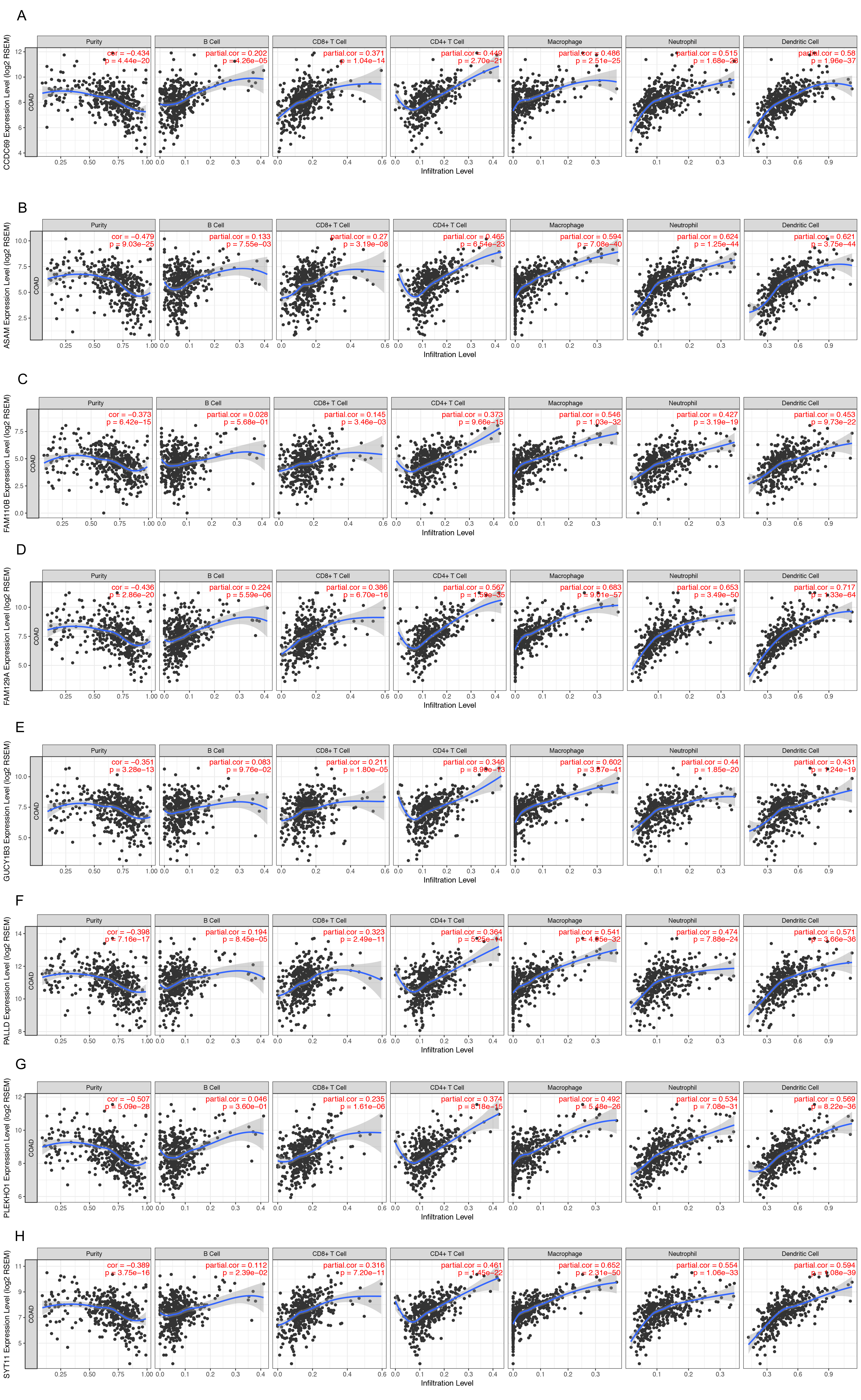

Supplement: Supplementary file 6 — Additional file 6: Figure S4 Validating the correlation between hub genes and immune cell infiltration. (A-H) We use the TIMER database to validate the correlation between the expression level of hub genes and the infiltration level of B cells, CD8+ cells, CD4+ cells, macrophages, neutrophils and dendritic cells in colon cancer tissues. The coefficient values and P values were calculated by Spearman coefficient. [file 12967_2020_2491_MOESM6_ESM.tif]

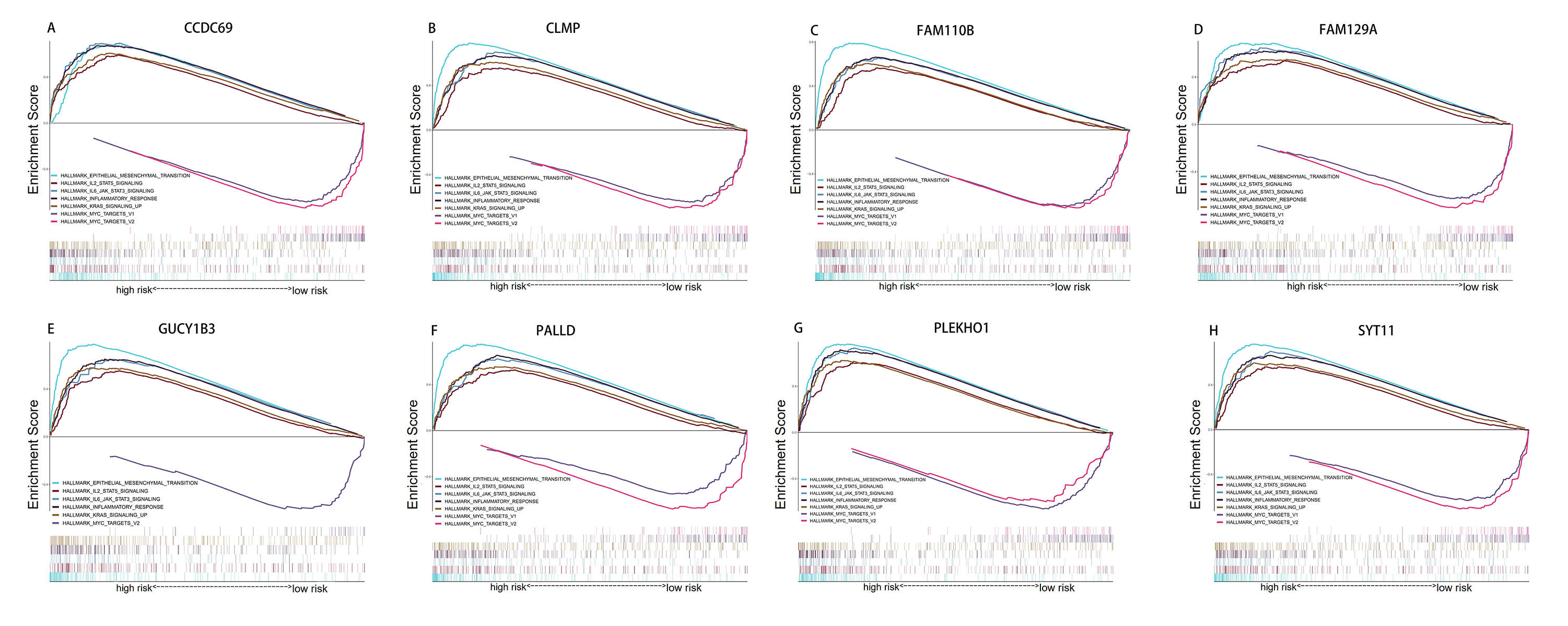

Supplement: Supplementary file 7 — Additional file 7: Figure S5 GSEA of hub genes. (A-H) The high-expression of most hub genes were enriched with epithelial mesenchymal transition, IL2-STAT signaling, IL6-JAK-STAT3 signaling, inflammatory response and KRAS signaling. The down-expression of these genes were enriched with MYC target V1 and V2. [file 12967_2020_2491_MOESM7_ESM.tif]
