# Supplementary material for: Analyzing and validating the prognostic value and mechanism of colon cancer immune microenvironment
Source: J Transl Med. 2020 Aug 28;18:324. doi: 10.1186/s12967-020-02491-w (PMC7456375; doi:10.1186/s12967-020-02491-w)
Supplement: Supplementary file 4 — Additional file 4: Table S1 The list of genes in the brown module. [file 12967_2020_2491_MOESM4_ESM.docx]

**Table S1 The list of genes in the brown module.**

| Genes in the brown module |
| --- |
| AKT3 |
| ENOX1 |
| LONRF2 |
| CNN1 |
| TCEAL2 |
| MAP6 |
| FGF7 |
| RASL12 |
| NEGR1 |
| TMEM200B |
| HAND2 |
| GLI3 |
| A2M |
| RBMS3 |
| ATP1A2 |
| EPHA6 |
| PDLIM3 |
| PCP4L1 |
| SLIT2 |
| FBXL7 |
| CALD1 |
| MAP1A |
| PGM5 |
| STUM |
| SORBS1 |
| GNAO1 |
| KCNMB1 |
| PSD |
| ZNF667 |
| LAYN |
| OR51E2 |
| PLA2G5 |
| JAM2 |
| ABCC9 |
| PHYHIP |
| RGMA |
| C20orf194 |
| MYH11 |
| FAM110B |
| RNF150 |
| ADAMTSL3 |
| SGCA |
| CSDC2 |
| DZIP1 |
| CLIP3 |
| SPG20 |
| SYNC |
| CHRDL1 |
| PKD2 |
| CASQ2 |
| PABPC5 |
| DACT3 |
| ACKR1 |
| AGTR1 |
| FILIP1 |
| TACR2 |
| DAAM2 |
| MYCT1 |
| MYOM1 |
| CYYR1 |
| TCF4 |
| MYL9 |
| WSCD2 |
| MYOCD |
| DOK6 |
| MAMDC2 |
| BVES |
| SYPL2 |
| SMYD1 |
| CASQ1 |
| C1R |
| ANK2 |
| FEZ1 |
| PDE2A |
| FHL1 |
| VSTM4 |
| C2orf40 |
| RBPMS2 |
| GAS7 |
| C1QTNF7 |
| NEXN |
| NCAM2 |
| PLN |
| ASB2 |
| ACTG2 |
| FRRS1L |
| ZDHHC15 |
| FERMT2 |
| CAVIN2 |
| CLMP |
| JAM3 |
| CACNA2D1 |
| TRPC4 |
| DNAJB5 |
| LDB2 |
| PALLD |
| ARHGEF25 |
| GDF5 |
| CFL2 |
| CCDC69 |
| GPM6A |
| STON1 |
| TAGLN |
| MAP1B |
| TPM2 |
| HSPB7 |
| HSPB8 |
| REEP2 |
| SYNPO2 |
| GREM1 |
| GPRASP1 |
| PRDM6 |
| TSHZ3 |
| MASP1 |
| MRVI1 |
| DIXDC1 |
| MPDZ |
| MEIS1 |
| DPP6 |
| PRIMA1 |
| ANGPTL1 |
| C14orf132 |
| NECAB1 |
| HSPB6 |
| LRCH2 |
| FILIP1L |
| THSD7A |
| FBXL22 |
| KIAA1462 |
| BOC |
| ITGA7 |
| JAZF1 |
| RBFOX3 |
| NAP1L3 |
| SYNM |
| KCNAB1 |
| GJC1 |
| ZNF521 |
| DDR2 |
| FLRT2 |
| GREM2 |
| JPH2 |
| RUNX1T1 |
| TEK |
| CHRDL2 |
| CSRP1 |
| ADAMTS8 |
| EMCN |
| METTL24 |
| POPDC2 |
| MORN5 |
| SCN7A |
| LDB3 |
| AOC3 |
| BNC2 |
| HSD17B6 |
| PPP1R12B |
| C1S |
| ATP2B4 |
| PTPRM |
| TNS1 |
| MRGPRF |
| RNF180 |
| CCBE1 |
| KCNMA1 |
| TLL1 |
| MSRB3 |
| PYGM |
| FAM129A |
| FXYD6 |
| S1PR1 |
| SHE |
| SPARCL1 |
| PEG3 |
| ZFHX4 |
| KANK2 |
| MAB21L2 |
| FLNC |
| SCRG1 |
| CAND2 |
| PLP1 |
| DES |
| FBXO32 |
| AMOTL1 |
| PDZRN4 |
| CHRNA3 |
| GUCY1B3 |
| ZEB1 |
| SORCS1 |
| SPEG |
| GUCY1A3 |
| ELOVL4 |
| PGR |
| CPEB1 |
| CNTNAP1 |
| PREX2 |
| LMOD1 |
| CHRM2 |
| NOVA1 |
| MFAP4 |
| RHOJ |
| ACTA2 |
| SLC2A4 |
| ITGB3 |
| SGCD |
| DPYSL3 |
| C8orf88 |
| SVIL |
| SYT11 |
| MYLK |
| KIAA1644 |
| MYOC |
