# Supplementary material for: Analyzing and validating the prognostic value and mechanism of colon cancer immune microenvironment
Source: J Transl Med. 2020 Aug 28;18:324. doi: 10.1186/s12967-020-02491-w (PMC7456375; doi:10.1186/s12967-020-02491-w)
Supplement: Supplementary file 5 — Additional file 5: Table S2 The GS and MM values of hub genes. [file 12967_2020_2491_MOESM5_ESM.docx]

**Table S2 The GS and MM values of hub genes.**

| Gene | MM | GS |
| --- | --- | --- |
| FAM110B | 0.809215 | 0.31971 |
| CLMP | 0.804517 | 0.364989 |
| PALLD | 0.834248 | 0.324267 |
| CCDC69 | 0.858664 | 0.357855 |
| FAM129A | 0.847606 | 0.320214 |
| GUCY1B3 | 0.833251 | 0.300879 |
| PLEKHO1 | 0.804782 | 0.516774 |
| SYT11 | 0.825902 | 0.391751 |
